# Prediction of pathologic complete response after single-dose MR-guided partial breast irradiation in low-risk breast cancer patients: the ABLATIVE-2 trial—a study protocol

**DOI:** 10.1186/s12885-023-10910-6

**Published:** 2023-05-09

**Authors:** Yasmin A. Civil, Arlene L. Oei, Katya M. Duvivier, Nina Bijker, Philip Meijnen, Lorraine Donkers, Sonja Verheijen, Zdenko van Kesteren, Miguel A. Palacios, Laura J. Schijf, Ellis Barbé, Inge R. H. M. Konings, C. Willemien Menke -van der Houven van Oordt, Paulien G. Westhoff, Hanneke J. M. Meijer, Gwen M. P. Diepenhorst, Victor Thijssen, Florent Mouliere, Berend J. Slotman, Susanne van der Velde, H. J. G. Desirée van den Bongard

**Affiliations:** 1 Department of Radiation Oncology, Amsterdam UMC Location Vrije Universiteit Amsterdam, Boelelaan 1117, Amsterdam, The Netherlands; 2 Cancer Center Amsterdam, Cancer Treatment and Quality of Life, Amsterdam, The Netherlands; 3 Laboratory for Experimental Oncology and Radiobiology (LEXOR), Center for Experimental Molecular Medicine (CEMM), Amsterdam UMC Location University of Amsterdam, Meibergdreef 9, Amsterdam, The Netherlands; 4 Cancer Center Amsterdam, Cancer Biology and Immunology, Amsterdam, The Netherlands; 5 Department of Radiation Oncology, Amsterdam UMC Location Universiteit van Amsterdam, Meibergdreef 9, Amsterdam, The Netherlands; 6 Department of Radiology, Amsterdam UMC Location Vrije Universiteit Amsterdam, Boelelaan 1117, Amsterdam, The Netherlands; 7 Department of Pathology, Amsterdam UMC Location Vrije Universiteit Amsterdam, Boelelaan 1117, Amsterdam, The Netherlands; 8 Department of Medical Oncology, Amsterdam UMC Location Vrije Universiteit Amsterdam, Boelelaan 1117, Amsterdam, The Netherlands; 9 Department of Radiation Oncology, Radboud University Medical Center, Geert Grooteplein Zuid 10, Nijmegen, The Netherlands; 10 Department of Surgery, Amsterdam UMC Location Vrije Universiteit Amsterdam, Boelelaan 1117, Amsterdam, The Netherlands

**Keywords:** Low-risk breast cancer, Ablative, Partial breast irradiation, MR-guided radiotherapy, Single-dose pre-operative radiotherapy, Stereotactic body radiation therapy, Pathologic response, Radiologic response, Toxicity, Cosmetic outcome

## Abstract

**Background:**

Partial breast irradiation (PBI) is standard of care in low-risk breast cancer patients after breast-conserving surgery (BCS). Pre-operative PBI can result in tumor downstaging and more precise target definition possibly resulting in less treatment-related toxicity. This study aims to assess the pathologic complete response (pCR) rate one year after MR-guided single-dose pre-operative PBI in low-risk breast cancer patients.

**Methods:**

The ABLATIVE-2 trial is a multicenter prospective single-arm trial using single-dose ablative PBI in low-risk breast cancer patients. Patients ≥ 50 years with non-lobular invasive breast cancer ≤ 2 cm, grade 1 or 2, estrogen receptor-positive, HER2-negative, and tumor-negative sentinel node procedure are eligible. A total of 100 patients will be enrolled. PBI treatment planning will be performed using a radiotherapy planning CT and -MRI in treatment position. The treatment delivery will take place on a conventional or MR-guided linear accelerator. The prescribed radiotherapy dose is a single dose of 20 Gy to the tumor, and 15 Gy to the 2 cm of breast tissue surrounding the tumor. Follow-up MRIs, scheduled at baseline, 2 weeks, 3, 6, 9, and 12 months after PBI, are combined with liquid biopsies to identify biomarkers for pCR prediction. BCS will be performed 12 months after radiotherapy or after 6 months, if MRI does not show a radiologic complete response. The primary endpoint is the pCR rate after PBI. Secondary endpoints are radiologic response, toxicity, quality of life, cosmetic outcome, patient distress, oncological outcomes, and the evaluation of biomarkers in liquid biopsies and tumor tissue. Patients will be followed up to 10 years after radiation therapy.

**Discussion:**

This trial will investigate the pathological tumor response after pre-operative single-dose PBI after 12 months in patients with low-risk breast cancer. In comparison with previous trial outcomes, a longer interval between PBI and BCS of 12 months is expected to increase the pCR rate of 42% after 6–8 months. In addition, response monitoring using MRI and biomarkers will help to predict pCR. Accurate pCR prediction will allow omission of surgery in future patients.

**Trial registration:**

The trial was registered prospectively on April 28th 2022 at clinicaltrials.gov (NCT05350722).

## Background

Over the last decades, the introduction of breast cancer screening and the implementation of digital mammography and MRI have resulted in an increased incidence of early-stage breast cancer. The incidence will continue to increase due to the growth and aging of the population and the increasing prevalence of risk factors across the world [1, 2]. Additionally, by scaling up traditional treatment and imaging modalities, the global 5-year net survival is estimated to rise from 67.9% to 78.2% [3]. Both trends result in an ongoing increase in the absolute number of breast cancer patients, for example from 3.9 million in 2019 to an expected 5.0 million in 2030 in the United States [4]. At the same time, there is a shift towards personalized and less invasive therapies aiming to decrease treatment-associated toxicity and to improve cosmetic results and quality of life of patients without compromising oncological safety. The most applied standard treatment for early-stage breast cancer is breast-conserving surgery (BCS) followed by whole breast irradiation (WBI) [5, 6]. However, the majority of ipsilateral breast tumor recurrences (IBTR) occur at or in the vicinity of the primary tumor site [7]. Therefore, partial breast irradiation (PBI), in which only the tumor bed is irradiated, has recently become standard treatment for low-risk patients [8]. In general, patients are eligible for PBI when they meet the American Society of Radiation Oncology (ASTRO) or the European Society for Radiation Oncology (ESTRO) suitable criteria [9, 10].

Postoperative PBI leads to a smaller irradiated breast volume, reduced radiotherapy-associated toxicity, and similar 5-year (0.5% vs. 1.1%) and 10 year (3.7% vs. 2.5%) IBTR rates compared to WBI [11, 12]. Nevertheless, postoperative PBI remains challenging as delineation of the tumor bed can be difficult, due to distortion of the breast and postoperative seroma in the surgical cavity, resulting in unnecessarily large irradiated volumes. In contrast to postoperative tumor bed delineation, pre-operative tumor delineation allows more precise target delineation with less interobserver variation between radiation oncologists, and smaller irradiated volumes since the tumor is still in situ [13–16]. As a result, radiotherapy-associated toxicity could be reduced and quality of life improved. Additionally, pre-operative PBI allows a higher radiotherapy (RT) dose per fraction due to the smaller target volume, thereby allowing ultra-hypofractionation to ultimately one single dose, and reducing treatment burden.

Pre-operative external beam PBI has previously been investigated in patients with low-risk breast cancer [17–24]. These studies showed excellent oncological outcomes with local recurrence in 0–3% of the patients and overall survival of 97–100% after a follow-up of 21 to 60 months [19, 21, 23]. Acute and late toxicity was mild to moderate in the majority of patients [17, 19–23]. Cosmetic outcomes reported by both patients and physicians were good to excellent in respectively 78–100% and 89–100% of the patients [19–23]. In the ABLATIVE study (NCT02316561), which preceded the current study, pathologic complete response (pCR) was achieved in 42% of the patients at 6 to 8 months after single-dose ablative pre-operative PBI [21]. This was higher compared to other studies that evaluated pCR after single-dose pre-operative PBI, potentially due to the longer interval between RT and BCS [17, 18, 20, 25].

Response monitoring using MRI is standard clinical practice in patients treated with pre-operative systemic therapy [26, 27]. After pre-operative RT, data on radiologic response on MRI or other imaging are limited. Two studies showed that a radiologic complete response (rCR) on MRI had a positive predictive value (PPV) for pCR of 67–88% and negative predictive value (NPV) of 76–85% after pre-operative PBI [21, 28]. This means that a significant proportion of patients with rCR on MRI still have residual tumor in the surgical specimen. In the ABLATIVE trial, 67% of patients with rCR had a pCR and 33% had near pCR after 6 to 8 months [21]. Additionally, the prediction of pCR after pre-operative systemic therapy using liquid biopsies and biomarkers in tumor tissue, such as tumor-infiltrating lymphocytes (TILs), have been evaluated, and to a much lesser extent after pre-operative RT [21, 29–31]. The ABLATIVE trial showed presence of TILs pre-irradiation and 6 to 8 months post-irradiation [29]. No significant difference in the number of pre-irradiation TILs was observed in responders and non-responders treated with pre-operative PBI. Consequently, more data is needed to establish the predictive value of biomarkers and to develop prediction models for pathologic response in patients treated with pre-operative RT. If pCR can be more accurately predicted after pre-operative PBI in the future, surgery could be omitted in these low-risk patients. In future patients without a pCR, single-dose pre-operative PBI could replace multiple fractionated post-operative radiotherapy, and tumor downstaging could lead to excision of less healthy breast tissue.

The ABLATIVE-2 trial aims to assess the rate of pCR in low-risk breast cancer patients treated with MR-guided single-dose pre-operative PBI. In addition, radiologic response on MRI, toxicity, oncological outcomes, cosmetic outcomes, quality of life, liquid biopsies, and immune response markers in blood and tumor tissue will be evaluated before and after pre-operative PBI.

## Methods/design

### Study design

The ABLATIVE-2 trial is a Dutch multicenter, phase II, single-arm prospective study at the Radiotherapy Departments of the Amsterdam University Medical Centres (UMC) and Radboudumc in the Netherlands. Eligible patients are treated with MR-guided pre-operative PBI. BCS will be performed at 12 months post-PBI. Between RT and surgery, tumor response is monitored using MRI at 2 weeks, 3, 6, 9 and 12 months, and compared with the baseline MRI (before PBI). In case of tumor progression on MRI at any time or when residual tumor at 6 months is suspected, BCS is performed immediately (within two to four weeks). The primary objective of this study is to determine the rate of pCR one year after single-dose ablative RT and BCS in patients with early-stage breast cancer. The secondary objectives are to evaluate the radiologic response on MRI, radiotherapy- and surgery-induced toxicity, cosmetic outcome, and patients’ quality of life and distress. The oncological outcomes: local, regional, and distant relapse rates and overall survival will be assessed. Liquid biopsies and immune response markers in blood and tumor tissue pre- and post-RT will be evaluated. All tumor tissue and blood samples of patients will be preserved at the Amsterdam UMC Central Biobank, location Vrije Universiteit Medical Center (VUmc).

### Study population

Patients are eligible if they have histologically confirmed invasive breast cancer with low-risk characteristics according to the suitable criteria of the ASTRO guidelines for PBI [9, 10]. Low-risk criteria in this trial are women of 50 years or older with a cT1N0 non-lobular tumor, Bloom and Richardson grade 1 or 2, hormone receptor-positive, and HER2-negative. The inclusion and exclusion criteria are summarized in Table 1 [9].

**Table 1:** Inclusion and exclusion criteria for the ABLATIVE-2 trial

Inclusion criteria	Exclusion Criteria
World Health Organization performance status 0–2	Legal incapacity
Females ≥ 50 years with a unifocal cT1N0 tumor	Breast cancer mutation gene carrier
Tumor histology as assessed on biopsy:	Distant metastasis
- Bloom-Richardson grade 1 or 2	Previous history of breast cancer or DCIS
- Non-lobular invasive histological type carcinoma	Another type of malignancy within 5 years before breast cancer diagnosis^a^
- Estrogen receptor positivity - HER2 receptor negativity	Signs of extensive ductal carcinoma in situ on histological biopsy or imaging
Tumor-negative sentinel node	MRI contra-indication
Adequate understanding of the Dutch language	Collagen synthesis disease
	Indication for treatment with (neo-)adjuvant chemotherapy
	Non-feasible dosimetric plan

^a^For patients with adequately treated carcinoma in situ of the cervix or basal cell carcinoma of the skin no specific time to the breast cancer diagnosis is required for inclusion. DCIS ductal carcinoma in situ

### Study outcomes

**Fig. 1:**
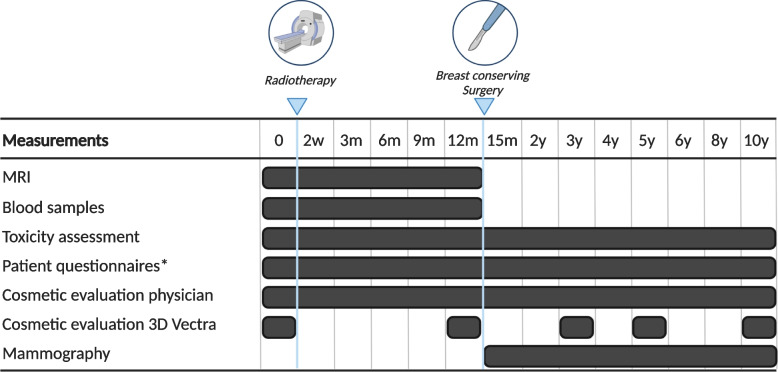
Overview of outcome measurements. Time points counted from radiotherapy treatment. Legend: w weeks, m months, y years. *questionnaires include evaluation of cosmetic outcomes and quality of life

The primary endpoint is the rate of patients with pCR one year after single-dose pre-operative RT treatment. Secondary endpoints are the time to rCR on MRI and the correlation between radiologic and pathologic response. In addition, radiotherapy- and surgery-induced toxicity will be assessed according to the Common Toxicity Criteria Adverse Events version 5.0 [32]. Patient-reported outcome measures will be assessed using the European Organization for Research and Treatment of Cancer core-30 (EORTC-QLQ-C30) and breast cancer-specific (QLQ-BR23) quality of life questionnaires and the Hospital Anxiety and Depression Scale (HADS) questionnaire. Cosmetic outcome is assessed by the patient using the BREAST-Q questionnaire. The radiation oncologist rates the cosmetic outcome as excellent, good, fair, and poor, based on breast changes such as telangiectasia and fibrosis. Cosmetic evaluation is also performed objectively using imaging captured by the VECTRA XT 3D-imaging system (Canfueld Sci, New Jersey, USA). Oncological outcomes are assessed using local, regional and distant relapse rates, and disease-free and overall survival since RT. Additionally, liquid biopsies and radiotherapy-associated immune response markers in blood and pre- and post-RT tumor tissue are investigated. The time points for the evaluation of outcomes are displayed in Fig. 1.

### Procedures

**Fig. 2:**
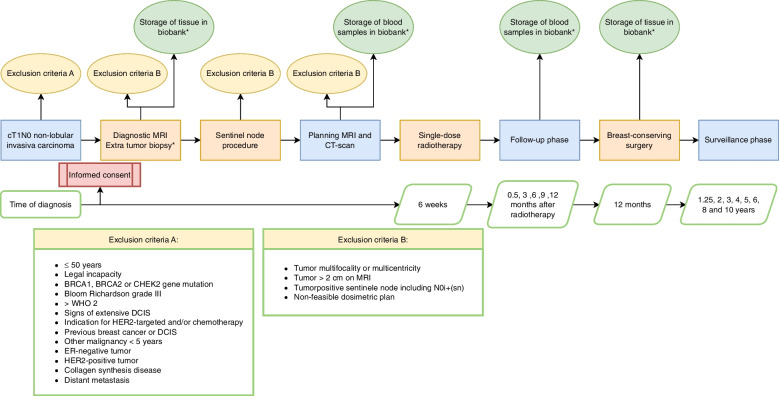
Overview study design. *Additional consent required

In Fig. 2 an overview of all study procedures in the ABLATIVE-2 trial is shown. The dedicated breast surgeon or breast cancer nurse will inform the patient during consultation on the possibility of participation in a trial evaluating pre-operative single-dose RT with delayed surgery. If the patient is interested in trial participation, written study information is handed over and additional information is given by the physician-researcher. This is followed by a referral for pre-operative consultation with the dedicated breast radiation oncologist.

#### Diagnostic workup

After informed consent, several procedures are required to confirm that the patient meets the eligibility criteria. First, a diagnostic MRI with perfusion and diffusion sequences in prone position is performed to exclude tumor multifocality or multicentricity and to assess tumor diameter. If no marker has been inserted in the tumor during the diagnostic biopsy, the radiologist will place an MRI-compatible marker in the tumor. Since pre-operative treatment will lead to tumor downstaging, a marker is mandatory for tumor localization during excision. After separate additional consent, extra tumor biopsies will be performed and stored in the Biobank. Secondly, the surgeon will perform a sentinel node procedure using technetium-99 m-nanocolloid, to rule out nodal involvement.

#### Treatment planning

Besides the standard radiotherapy planning CT in the treatment position, a planning MRI scan will be performed in treatment position. The gross tumor volume (GTV), which is the breast tumor on the diagnostic MRI, will be delineated on the planning MRI by a radiation oncologist specialized in breast cancer. GTV delineation is verified by a dedicated breast radiologist. To account for microscopic disease, the GTV is uniformly expanded by 2 cm to create the clinical target volume (CTV), while excluding the first 5 mm below the skin and the entire chest wall including the pectoral muscles, and not extending outside the breast tissue. For generating the planning target volumes (PTV), the GTV and CTV are expanded by 3 mm to obtain the planning target volumes PTV_GTV_ and PTV_CTV_, respectively. The PTV is a margin to account for patients’ movements during treatment and setup uncertainties.

**Fig. 3:**
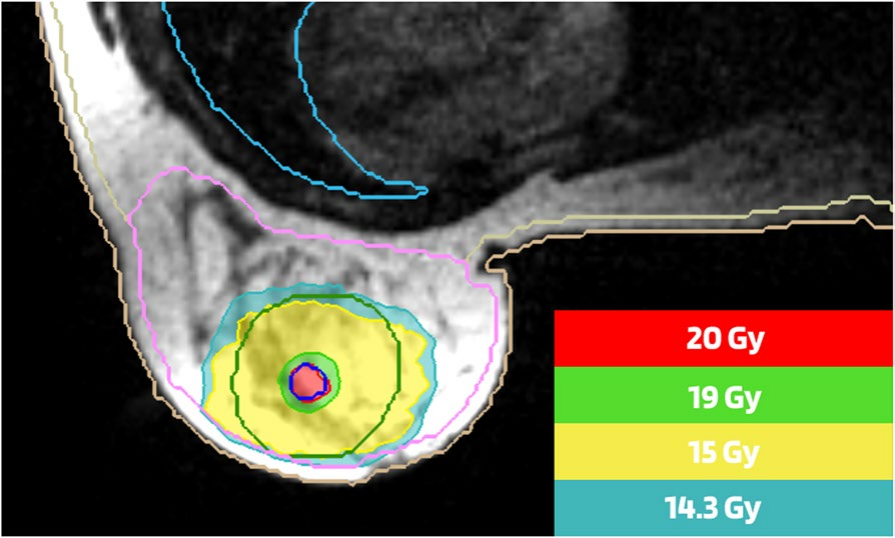
Dose distribution of single-dose ablative radiotherapy on MRIdian (Viewray, USA). Legend: The red isodose (20 Gy) represents the prescribed dose to the gross tumor volume (GTV). The green isodose (19 Gy) represents 95% of the prescribed dose to the GTV. The yellow isodose (15 Gy) represents the prescribed dose to the clinical target volume (CTV). The blue isodose (14.3 Gy) represents 95% of the prescribed dose to the CTV

For MR-guided radiotherapy, intensity modulated radiotherapy (IMRT) technique will be used on the MRIdian (ViewRay, USA) or Elekta Unity (Elekta, Sweden). On the conventional linac, IMRT or volumetric modulated arc therapy (VMAT) techniques will be used. In a single fraction, two RT dose levels are concomitantly prescribed: 15 Gy to the PTV_CTV_ and 20 Gy to the PTV_GTV_. Adequate target coverage is defined as a Dmean of the PTV_GTV and_ PTV_CTV_ of 99–101%, 98% or more of the PTVs receiving at least 95% of the prescribed dose and 2% or less of the PTVs receiving 107% of the prescribed dose, whilst respecting the organs at risk (OAR) doses (Table2). If optimal target volume cannot be achieved without exceeding the predefined dosimetrical constraints, the patient will be excluded from the study. In Fig. 3 an example of a single-dose PBI treatment plan is illustrated.

**Table 2:** Dose constraints for organs at risk in the ABLATIVE-2 trial

Heart	V_2.8 Gy_ < 10%
	V_4.7 Gy_ < 5%
	D_mean_ ≤ 1.2 Gy
	V_16Gy_ < 15 cc
Lungs	D_mean_ ≤ 2.66 Gy
Ipsilateral lung	V_6.2 Gy_ < 10%
	V_12.4 Gy_ < 0.5 cc
Chest wall	D_20cc_ < 16 Gy
	V_20Gy_ < 1 cc
Skin	Dose as low as possible; aim for D_0_._1 cc_ < 12 Gy if this is not feasible aim for D_0_._1 cc_ < 16 Gy
Optional dose constraints	
Ipsilateral breast	D_mean_ < 5 Gy
	PTV_CTV < 25% of total ipsilateral breast volume
Contralateral breast	No constraint, dose as low as possible

#### Radiotherapy treatment delivery

The single-dose PBI will be delivered within three weeks following the planning MRI- and CT scan. On the conventional linacs and the MRIdian (ViewRay Inc., USA), MR-guided RT delivery will take place either with repeated breath-hold periods for patients with significant tumor movement or in free breathing when the tumor position is not affected by the breathing movement. On the Elekta Unity (Elekta, Sweden), treatment delivery will be performed using free-breathing. On both MR-linacs, online adaption of the treatment plan will be performed based on the MRI immediately before treatment delivery. Real-time MRI tumor tracking will be used on the MRIdian during the treatment procedure. On the conventional linacs, real-time position management and/or surface guidance will be used for patient tracking. The patient will fill out a questionnaire on comfort during the treatment procedure.

#### Follow-up after single-dose ablative treatment

Frequent clinical and radiological evaluation will be performed after single-dose PBI at 2 weeks, 3, 6, 9, and 12 months. Tumor response will be classified according to the ‘Response Evaluation Criteria in Solid Tumors’ guidelines [33]. Radiologic complete response on MRI is defined as the complete absence of pathologic contrast enhancement and complete absence of pathologic apparent diffusion coefficient reduction in the original tumor bed. Additionally, at baseline and during each follow-up consultation (i.e. at 2 weeks, 3, 6, 9, and 12 months) blood samples will be taken.

Patients with an indication for endocrine therapy will start this treatment following RT. The treating medical oncologist will monitor the endocrine treatment. If the surgical specimen shows any microscopic findings resulting in an indication for the start or change of adjuvant systemic therapy according to the Dutch national guidelines, patients will be treated accordingly [34].

#### Breast-conserving surgery

To assess the effect of RT on the breast tumor, BCS is performed at 12 months following RT. BCS will be performed ahead of time in case radiologic tumor progression (an increased size of contrast enhancement) is observed, or in case the MRI does not show rCR at 6 months following RT.

The surgical specimen is evaluated by a dedicated breast pathologist at the hospital where the surgery is performed. Viable tumor cells will be evaluated using hematoxylin and eosin staining and the activity of cytokeratin antibodies. Pathologic response will be categorized according to the European Society of Mastology (EUSOMA criteria).

- 1. Complete pathologic response is defined as either no residual carcinoma or no residual invasive carcinoma but ductal carcinoma in situ (DCIS) may be present.
- 2. Partial response to therapy
- a. Near complete response is defined as minimal residual disease (<10% tumor cells)
- b. Evidence of response (10-50% tumor cells)
- c. >50% tumor cellularity remains evident with features of response present (e.g. fibrosis)
- 3. No evidence of response

In addition, the proportion of patients with DCIS in the surgical specimen will be recorded. The surface of the excision specimen will be marked with Indian ink to evaluate surgical margins. Surgical margins will be described as minimal microscopic tumor-free margins of invasive and in situ carcinoma in millimeters (mm). This will always be performed in the direction of the nearest surgical margin. If tumor-positive margins are found, a radical re-excision needs to be performed.

#### Follow-up

Clinical consultations with the treating radiation oncologist including physical examination will be performed yearly from BCS to 5 years after single-dose RT, and every other year from 6 to 10 years. During the consultation, toxicity and cosmetic outcome will be assessed by the radiation oncologist (Fig. 1). Questionnaires on quality of life, distress, and cosmetic outcome questionnaires will be filled out by the patient. Digital photographs for objective evaluation of cosmetic outcome will also be taken at 3, 5, and 10 years after RT. As per standard of care, radiological follow-up will consist of yearly mammograms in the first 5 years. After 5 years, additional mammograms will be performed at 6, 8, and 10 years after RT.

### Biobank

The diagnostic biopsy, the surgical specimen, and blood samples will be preserved for 25 years at the Amsterdam UMC Central Biobank, location VUmc for future research in the field of breast cancer if patients give additional consent. To evaluate the radiation effect on gene expression in the breast tumor, additional consent for gene expression profiling is obtained. Since the tissue volume of the diagnostic biopsy is too little for gene expression profiling, 2–3 extra 14G tumor biopsies are taken after additional informed consent by the patient. RNA is easily degraded during the process of formalin fixation and a large variety exists due to different protocols used for fixation [35, 36]. Therefore, fresh frozen biopsies are used for gene expression profiling with RNA.

### Ethical aspects

This study is conducted according to the Declaration of Helsinki (Fortaleza, Brazil, October 2013) and the Dutch Medical Research Involving Human Subjects Act (http://www.ccmo.nl). The study protocol has been approved by the Medical Research Ethic Committee of the Amsterdam UMC, location VUmc (NL77000.029.21), and has been registered at an international trial registry (ClinicalTrial.gov: NCT05350722). The study has been approved by the Institutional Review Board of each participating center. After a written and oral explanation of the study, all patients are required to give written informed consent before inclusion.

### Quality assurance

To assure the quality and validity of the research data, an independent qualified monitor will carry out study monitoring centrally at the Clinical Monitoring Center of the Amsterdam UMC, location VUmc. The monitoring will be performed according to national guidelines on quality control for Dutch University Medical Centers [37].

### Statistical analysis

Sample size calculation is performed based on the primary endpoint: the rate of pathologic complete response. We expect that 40% of our patients will achieve pathologic complete response 12 months after high-dose single fraction PBI. The sample size calculation is performed using the Wilson method for the calculation of different 95% confidence intervals (CI) for proposed sample sizes. A sample size of 100 patients would produce an acceptable 95% CI ranging from 30.9% to 49.8% while maintaining practical applicability.

The proportion of patients with pCR 12 months after RT will be evaluated and a 95% CI will be calculated. Regarding the secondary outcome, radiologic response, the negative and positive predictive value of radiologic response for pCR with 95% CIs will be calculated for all intervals between RT and BCS. Patient-reported and cosmetic outcomes will be analyzed in a linear mixed model for repeated measures. Radiation- and surgery-induced toxicity, local, regional and distant relapse rates, disease-free and overall survival will be reported using descriptive statistics. Kaplan–Meier method will be used to quantify disease-free and overall survival. The natural variability of the response markers will be evaluated for all accrued patients, exploring overlap in variance among the different response markers, and if applicable studying the reproducibility of the potential response markers.

## Discussion

The ABLATIVE-2 study is a continuation of the ABLATIVE study (NCT02316561) in which 36 patients were treated with a single ablative RT dose and BCS at 6 or 8 months following RT. This study showed that single-dose pre-operative PBI was technically and clinically feasible in patients with low-risk breast cancer [21]. The pCR rate after 8 months between RT and BCS seemed to be higher than after 6 months (48% vs. 33%). Therefore, in the current ABLATIVE-2 design, the interval between RT and BCS is prolonged to 12 months. In addition, the predictive value of quantitative MRI parameters and immunological markers, and potential biomarkers in blood and tumor tissue for pCR will be evaluated.

Over the last decades, clinical research has been focused on personalized treatment aiming to de-escalate breast cancer treatment. In patients with early-stage breast cancer, BCS followed by WBI resulted in equivalent recurrence rates and overall survival compared with mastectomy [5, 38, 39]. In addition, axillary RT has replaced axillary lymph node dissection in patients with a tumor-positive sentinel node [40]. Genomic tools are utilized to identify low-risk patients with no benefit from chemotherapy [41, 42]. Irradiation times of breast cancer patients have been shortened with (ultra-) hypofractionated radiation schedules and treatment volumes have been reduced with accelerated PBI instead of whole breast irradiation in low-risk patients [11, 12, 43–46]. Thus, breast cancer treatment has become increasingly patient-tailored over the years.

Improved diagnostic modalities and treatment have led to a 15% decline in breast cancer mortality rates in Europe between 2002 and 2012 [47]. However, breast cancer patients seem to prefer treatments that result in less functional disability and pain over progression-free survival months [48]. As such, studies focusing on reducing radiation- and surgery-induced toxicity and improving the cosmetic outcome of personalized breast cancer treatment are increasingly performed. The introduction of pre-operative PBI has been gaining interest and has the potential to lead to a paradigm shift in the treatment of low-risk patients [17–23, 49]. PBI in the pre-operative setting has several advantages over post-operative treatment. Pre-operative tumor delineation is more accurate and leads to a reduced volume of irradiated healthy breast tissue, while still adhering to oncologically safe margins [13–15]. Precise irradiation could lead to milder toxicity and better cosmetic outcome [50]. The tumoricidal effect of irradiation can lead to tumor downstaging and preservation of breast tissue during surgical excision. In the ABLATIVE study an higher pCR rate of 42% was observed after a longer interval between pre-operative radiotherapy and surgery of 6 to 8 months [21]. In the PAPBI trial, 133 patients were treated with pre-operative PBI. Seventy-eight patients were treated with 40 Gy in 10 fractions in 2 weeks and 55 patients with 30 Gy in 5 fractions in 1 week [19]. BCS was performed 6 weeks after pre-operative PBI and a pCR rate of 10% was reported. IBTR was found in 3% of the patients after a median follow-up of 5 years and overall survival was 97%. The study by Nichols et al. included 27 patients, who received pre-operative PBI 10 × 3.85 Gy in 1 week [20]. After 3 weeks, pCR was achieved in 15% of the patients. Toxicity was mainly mild in this study and good to excellent cosmetic outcome was reported by 78% of the patients. Similar results were found in the ROCK trial. Twenty-two patients were treated with a pre-operative single-dose of 21 Gy using the Cyberknife® and BCS after 2 weeks [24]. Two patients (9%) experienced a pCR and no toxicity higher than grade 2 was reported. Cosmetic outcomes on the other hand were rated as good or excellent by 62% of the physicians. The SPORT-DS trial evaluated a pre-operative single-dose of 20 Gy and another retrospective cohort study evaluated a schedule with multiple fractions (3 × 9.5 Gy) followed by BCS after 3 and 6–8 weeks, respectively [17, 18]. These studies did not report a pCR in any of the patients, despite similar interval durations between PBI and BCS compared to the previously described studies. This difference could be explained by the small sample sizes.

The post-operative complication rate of BCS after pre-operative PBI is 14–17% [19, 21]. These complications include hemorrhage and wound infection. Analysis of 648 patients in the Cambridge IMRT trial, in which patients were treated with BCS followed by WBI, showed a post-operative infection rate of 19.7% and a hematoma rate of 7.9% [51]. Results of the study have proven that the presence of seroma is associated with post-operative infection and hemorrhage [52]. The FOCUS cohort study collected data on all consecutive patients aged 65 years and older with breast cancer in the Netherlands between 1997 and 2004 [53]. The post-operative complication rate for patients treated with BCS was 14%, which is similar to the patients treated with pre-operative PBI. In the ABLATIVE-2 trial, patients will have 12 months of recovery time until BCS is performed. So the negative effect of RT on the incidence of post-operative complications is expected to be minimalized.

Long-term outcomes of pre-operative PBI are awaited with the continuation of follow-up in these studies [17, 23, 54]. Continuation of patient accrual in the SPORT-DS trial will provide more information on tumor response rate in a larger study population (Table 3) [17]. The SIGNAL-2 trial (NCT02212860) will prolong the interval between RT and BCS from 1 to 3 weeks [22]. Based on the positive results of the previous phase I trial of Horton et al., a larger patient cohort will be treated with a single dose of 21 Gy at Duke University (NCT02482376) [23]. The optimal ablative dose has not yet been established, thus phase I dose-escalation trials (NCT04679454, NCT04040569) are recruiting patients to identify the maximum tolerated dose. The ABLATIVE-2 study is, to the best of our knowledge, the first study to evaluate the effect of pre-operative single-dose PBI after a longer interval, i.e. 12 months.

**Table 3:** Summary of the clinical trial regarding pre-operative partial breast irradiation

Trial ID, status	Title	Treatment	Primary endpoints	Secondary endpoints	Estimated primary completion date
NCT05350722, recruiting	Single-dose Preoperative Partial Breast Irradiation in Low-risk Breast Cancer Patients (ABLATIVE-2)	Pre-operative single-dose radiotherapy (20 Gy) and BCS after 12 months	Pathologic complete response	Radiologic complete response, treatment-related adverse events, quality of life, cosmetic outcome, oncological outcomes, immune response and biomarkers	March 2025
NCT02913729, recruiting	Pre- Versus Postoperative Accelerated Partial Breast Irradiation (PAPBI-2)	Pre vs. post-operative PBI (5 × 5.2 Gy) and BCS 6 months after PBI	Cosmetic outcome	Pathologic complete response, postoperative complications	December 2022
NCT03917498, active/not recruiting	Single Pre-Operative Radiation Therapy—With Delayed Surgery for Low Risk Breast Cancer (SPORT-DS)	Pre-operative single-dose radiotherapy and BCS after 3 months^a^	Pathologic complete response	Radiation toxicity	February 28, 2020 (actual)
NCT02212860, active/not recruiting	Stereotactic Image-Guided Neoadjuvant Ablative Radiation Then Lumpectomy (SIGNAL 2)	Pre-operative PBI (21 Gy or 3 × 10 Gy) and BCS after 14–20 days	Immune priming, angiogenesis, proliferation/hypoxia/apoptosis/invasion markers, toxicity	Cosmetic outcome, survival	April 2021 (actual)
NCT03520894, recruiting	Radiotherapy in Preoperative Setting With CyberKnife for Breast Cancer (ROCK)	Pre-operative single-dose radiotherapy (21 Gy) and BCS after 30 days	Acute skin toxicity	Pathologic complete response, rate of complete resection, oncological outcomes, chronic toxicity, differential genetic expression, immune response and biomarkers	May 1, 2022
NCT04679454, recruiting	Single Fraction Preoperative Radiotherapy for Early Stage Breast Cancer (CRYSTAL)	Pre-operative single dose radiotherapy (18 Gy, 21 Gy, 24 Gy) and BCS after 4–8 weeks	Dose escalation, pathologic complete response	Chronic toxicity, cosmetic outcome, postoperative complications, oncological outcomes	March 2026
NCT03909282, recruiting	Phase 2 Surgical Excision vs Neoadjuvant Radiotherapy + Delayed Surgical Excision of Ductal Carcinoma (NORDIS)	Pre-operative PBI (5 × 6 Gy) and BCS after 3 months vs. upfront surgery	Rate of DCIS pathologic complete response	Wound complication, correlation of imaging characteristics and pathologic findings, rate of invasive carcinoma	September 2024
NCT04040569, recruiting	A Phase I Dose Escalation Study of Single Fraction Pre-operative Stereotactic Partial Breast Irradiation (S-PBI) for Early Stage Breast Cancer	Pre-operative single dose radiotherapy (30 Gy, 34 Gy, 38 Gy) and BCS^b^	Dose escalation, cosmetic outcome	-	September 2024
NCT02482376, active/not recruiting	Preoperative Single-Fraction Radiotherapy in Early Stage Breast Cancer	Pre-operative single-dose radiotherapy (21 Gy) and BCS^b^	Physician reported cosmetic outcome	Ki-67, patient reported cosmetic outcome, gene expression, local control, circulating cell free DNA	March 2025

*BCS* Breast conserving surgery, *PBI* Partial breast irradiation

^a^Dose not reported

^b^Timing of surgery not specified

The prediction of pCR using MRI will be challenging in the current study design. MRI will be performed at different time points after RT to evaluate tumor response. The MRI at 2 weeks in particular aims to identify early radiation effects. The parameter extraction from an MRI contains substantial variability across patients [55]. Therefore, reference objects, image acquisition protocols, and software for image data analysis need to be implemented. Hence, study results will have to identify other radiological predictors or tumor response markers in blood or tumor tissue to select good responders to RT.

A meta-analysis showed that the detection rates of ctDNA in blood samples of patients with early-stage breast cancer at baseline range between 23–100% [31]. Most studies included patients with triple-negative breast cancer, who have a higher ctDNA detection rate than patients with an ER + tumor [31, 56–58]. In patients with ER + early-stage breast cancer, ctDNA was detected in 24% of the patients (*n* = 51) [58]. However, the median ctDNA level, measured using personalized digital polymerase chain reaction assays, was 0 copies/ml in patients with a clinical T1 tumor. The detection of ctDNA in patients with early-stage breast cancer increases with deep sequencing using large gene panels. Zhang et al. prospectively collected plasma samples of 102 early-stage breast cancer patients and reached a positive detection rate of 74% (49/66) [56]. For patients with a clinical T1 tumor, the detection rate was 56%. Patients with a higher number (40–90%) of TILs were more likely to have ctDNA detection (92%) compared to patients with a low number (< 10%) of TILs (60%).

Similar to ctDNA detection, TILs levels are higher in triple-negative and HER2 + tumors [59, 60]. Still, in patients with luminal A breast cancer TILs have a prognostic value [61, 62]. The SweBCG91cRT trial has shown that patients with a Luminal A tumor and a low TILs score have a 51% reduced risk of IBTR when treated with post-operative RT compared to no post-operative RT [61]. In the ABLATIVE study, six to eight months after single-dose pre-operative PBI, a decrease of CD3, CD4, and CD8 TILs was found in tumor tissue [29]. However, no differences in TILs levels were found in responders vs. non-responders. Due to low TILs levels in luminal A tumors and subtle differences after treatment, analyzing a larger patient cohort will be necessary to predict pCR after single-dose pre-operative PBI using TILs.

Post-operative endocrine therapy is the standard of care for patients with an indication according to the Dutch guidelines. As surgery is scheduled 6 to 12 months after RT, endocrine therapy is allowed after RT (and before surgery) in this trial, despite possible tumor downstaging. The ABLATIVE-2 study has intensive MRI follow-up to monitor tumor response after pre-operative PBI and allows safe prolongation of the interval to BCS. In prospect, the number of MRIs could be reduced in case no disease progression is observed during the monitoring phase in this trial. In addition, the extensive diagnostic workup including a sentinel node procedure could be de-escalated, if favorable oncological outcomes are found in the current SOUND trial (Sentinel Node vs Observation After Axillary Ultrasound, NCT02167490) of the European Institute of Oncology in which the omission of the sentinel node procedure in clinically node-negative cT1 breast cancer patients undergoing BCS is investigated. This will make the clinical implementation of pre-operative single-dose PBI more practical. Nonetheless, MR-guided RT delivery is not common practice in most hospitals. The use of conventional and MR-linacs from different manufacturers could influence clinical outcomes of radiation treatment due to variations in the treatment protocols of the machines. The MR-linac provides an online adaptive workflow with good visibility of the breast tumor on MRI. This allows more precise treatment delivery and irradiation of smaller non-intended breast volumes reducing radiation-induced toxicity.

The ABLATIVE-2 trial is a multicenter prospective trial and contributes to the advancement of tailored breast cancer treatment with pre-operative single-fraction PBI in patients with low-risk breast cancer. If tumor response markers in blood or tumor tissue and radiological parameters are identified to successfully predict pCR in future patients, BCS could potentially be omitted in future low-risk patients. When a patient does not reach pCR after pre-operative single-fraction PBI, downstaging of the tumor could reduce excision volumes of healthy breast tissue and the number of RT fractions could be reduced to one single dose instead of the post-operative standard schedule of multi-fractionated RT for patients with early-stage breast cancer [43]. This could result in an overall reduction of treatment burden for patients and improve logistic challenges in healthcare.

## Data Availability

Not applicable – data collection is still ongoing in this trial.

## Abbreviations

BCS: Breast conserving surgery
WBI: Whole breast irradiation
IBTR: Ipsilateral breast tumor recurrence
PBI: Partial breast irradiation
ASTRO: American Society of Radiation Oncology
ESTRO: European Society of Radiation Oncology
RT: Radiotherapy
pCR: Pathologic complete response
rCR: Radiologic complete response
PPV: Positive predictive value
NPV: Negative predictive value
TILs: Tumor-infiltrating lymphocytes
UMC: University Medical Centre
VUmc: Vrije Universiteit Medical Centre
EORTC-QLQ-C30: European Organization for Research and Treatment of Cancer core-30 questionnaire
EORTC-QLQ-BR23: European Organization for Research and Treatment of Cancer breast cancer-specific questionnaire
HADS: Hospital Anxiety and Depression Scale
Linac: Linear accelerator
GTV: Gross Tumor Volume
CTV: Clinical Target Volume
PTV: Planning Target Volume
IMRT: Intensity modulated radiotherapy
VMAT: Volumetric modulated arc therapy
OAR: Organs at risk
EUSOM: European Society of Mastology
DCIS: Ductal carcinoma in situ
mm: Millimeter
CI: Confidence Interval
